# Could flies explain the elusive epidemiology of campylobacteriosis?

**DOI:** 10.1186/1471-2334-5-11

**Published:** 2005-03-07

**Authors:** Karl Ekdahl, Bengt Normann, Yvonne Andersson

**Affiliations:** 1 Department of Epidemiology, Swedish Institute for Infectious Disease Control (SMI), SE-17182 Solna, Sweden; 2 Department of Medical Epidemiology and Biostatistics, Karolinska Institute, SE-17177 Stockholm, Sweden; 3 County Medical Office for Communicable Disease Control, SE-581 91 Linköping, Sweden

## Abstract

**Background:**

Unlike salmonellosis with well-known routes of transmission, the epidemiology of campylobacteriosis is still largely unclear. Known risk factors such as ingestion of contaminated food and water, direct contact with infected animals and outdoor swimming could at most only explain half the recorded cases.

**Discussion:**

We put forward the hypothesis that flies play a more important role in the transmission of the bacteria, than has previously been recognized. Factors supporting this hypothesis are: 1) the low infective dose of *Campylobacter*; 2) the ability of flies to function as mechanical vectors; 3) a ubiquitous presence of the bacteria in the environment; 4) a seasonality of the disease with summer peaks in temperate regions and a more evenly distribution over the year in the tropics; 5) an age pattern for campylobacteriosis in western travellers to the tropics suggesting other routes of transmission than food or water; and finally 6) very few family clusters.

**Summary:**

All the evidence in favour of the fly hypothesis is circumstantial and there may be alternative explanations to each of the findings supporting the hypothesis. However, in the absence of alternative explanations that could give better clues to the evasive epidemiology of *Campylobacter *infection, we believe it would be unwise to rule out flies as important mechanical vectors also of this disease.

## Background

*Campylobacter *infection is a zoonotic disease, observed in most parts of the world. The disease is caused by *Campylobacter jejuni*, or less commonly *Campylobacter coli*. It is estimated to cause 5–14% of diarrhoea, worldwide [1], and also in the Western world *Campylobacter *infection has emerged to be the most important bacterial cause of gastrointestinal infection. Animals (variety of fowl, swine, cattle, sheep, dogs, cats, and rodents) are the major reservoir for the bacteria. *Campylobacter *does not easily grow in food, but the critical infective dose is low [2]. Unlike salmonellosis with well-known routes of transmission, the epidemiology of campylobacteriosis is still largely unclear [3]. Known risk factors for the disease include ingestion of undercooked meat, contaminated food and water or raw milk, direct contact with pets, farm animals and small children, and swimming in lakes, but also travel abroad [2,4-6]. Direct person-to-person transmission between adults appears to be uncommon. In temperate regions, campylobacteriosis has a distinct seasonal pattern, with the peak incidence in the summer months [3,5,7,8]. Identified risk factors for *Campylobacter *infections, that may coincide with the summer peaks in the temperate regions include direct animal contact, eating barbecued meals, swimming in lakes, and drinking untreated water from streams and other natural sources [4-6,9]. However, all these factors could at most explain 50% of the sporadic cases [3]. Instead we put forward the hypothesis that flies play a more important role in the transmission of the bacteria, than has previously been recognized.

## Discussion

### The fly hypothesis

The common houseflies (*Musca domestica*) and other muscid flies thrive in excreta and other filth. They could act as mechanical vectors, by carrying bacteria on the hairs and surface of their bodies or on the glandular hairs on their feet, but they could also act as biological vectors by passage through the alimentary tract, where pathogens have opportunity to multiply [10]. The houseflies are important mechanical vectors in the transmission of many infectious diseases with low infective dose, such as shigellosis, typhoid fever and *E. coli *infection [11,12]. Fly control has shown to be effective in preventing childhood diarrhoea in Pakistan and The Gambia [13,14], and shigellosis in Israeli Army personnel [15]. Already in 1983, Rosef and Kapperud postulated that flies might play a linking role by transmitting *Campylobacter *from animals to human food [16]. Since then several researchers have unravelled the role of flies in the epidemiology of avian campylobacteriosis [17-19], but the idea of flies as important vectors for human *Campylobacter *infection has been largely neglected [20]. Six factors speak in favour of our hypothesis.

#### 1. Infective dose

The infective dose of *Campylobacter *can be as low as 800 bacteria [21], which is in the same magnitude as that of *Shigella *spp, *Salmonella *Typhi, and *E. coli*, pathogens that are known to be transmitted by flies [11,12,15], and much lower than the infective dose of *Vibrio cholerae *(10^8 ^bacteria), and non typhoidal *Salmonella *species (10^5^-10^10 ^bacteria). Although less tolerant to desiccation than some other food-borne pathogens [22], *Campylobacter *can survive on dry surfaces for at least seven days [23], thus enabling the bacteria to survive for several days both on the body of the fly and in desiccated fly faeces.

#### 2. Flies as a possible vector

Studies have shown that *Campylobacter *could easily be transmitted from the environment to flies [17,24], and thus making flies a reservoir for the bacteria. *Campylobacter *could also be transmitted from flies to chickens [19]. In a recent study, *Campylobacter *could be isolated from 4 of 49 (8%) of flies caught outside a broiler house in Denmark. Furthermore, Wright showed that *Campylobacter *could be isolated from five of 210 (2.4%) living flies, isolated from three different locations [25]. From these results the author drew the conclusion that the health hazard from the transmission of *Campylobacter *from animals to human food is small. On the contrary, giving the numerous contacts between flies and human food, we find it highly likely that if one out of every 40 flies carries *Campylobacter *the health hazard would be significant.

#### 3. Presence of the pathogen in the environment

*Shigella *is a strict human pathogen, while the major source of *Campylobacter *is the faeces of both humans and animals such as chickens, cattle and pigs, which are often kept in close proximity of humans. Stanley and Jones have previously shown the importance of cattle and sheep farms as reservoirs of *Campylobacter *[26]. *Campylobacter *is also common in the droppings from wild birds [27,28], and ubiquitous in the environment. *Campylobacter *spp have been isolated from sewage contaminated water [29], contaminated soil [30] and aquatic sediments [31], and in sand from bathing beaches [32]. There are therefore likely considerably more *Campylobacter *than *Shigella *in the close vicinity of humans. Since flies have been shown to be an important mechanical vector of shigellosis, it would be surprising if they could not also be so for campylobacteriosis. Direct transmission from the soil could probably account for some of the cases in children, but less likely for adult cases.

#### 4. Seasonality of the disease

The distinct seasonality in the temperate regions [3,5,7,8,33] fits well with the fly hypothesis. The summer is the only season in temperate countries when people are in close contact with flies – often while having picnics or otherwise eating outdoor in close proximity of cattle and other environmental sources of *Campylobacter*. Some of the recorded association between barbeque and campylobacteriosis could very well be due to contamination of the food by flies, rather than undercooked meat or cross-contaminations, as has previously been postulated. A recent study from the UK has shown a close temporal association between the incidence of campylobacteriosis and fly density [34]. Although there is a seasonal pattern in the density of flies in the tropics, flies are present year round [13,14]. Therefore, if our hypothesis holds true, there should not be the same distinct seasonal peaks in the tropics. However seasonal data on campylobacteriosis from tropical regions are largely lacking. Instead we have recently compared Swedish notification data on travel-related campylobacteriosis with an extensive database on the travel patterns of Swedish residents (denominator for monthly risks per region). While a distinct seasonal pattern, as previously described, could be discerned in travellers from all temperate regions, the risk of campylobacteriosis in travellers from the tropics were more dispersed over the year [35]. Lack of detailed data on seasonal fly density and quite large geographical regions for our risk estimates of campylobacteriosis, prevented us from making any correlations between risk of campylobacteriosis and the presence of flies in the tropical regions.

#### 5. Age profile

Small children are less able to protect themselves from flies than older children and adults, and are more likely to have their hands on fly-soiled surfaces. In the tropics, the *Campylobacter *infection is largely confined to children below the age of two years, and the decreasing incidence thereafter has been attributed to a lasting immunity [20]. On the contrary, in Sweden and other Western countries, the highest incidence is seen in young adults, with a smaller peak in pre-school age children [20,36]. Then, how about western travellers going to the tropics? If the major transmission route of *Campylobacter *was ingestion of contaminated food, one would expect the infection to be relatively evenly distributed among the largely non-immune westerners coming to high prevalence countries. Again we turned to the risk estimates for campylobacteriosis in returning Swedish travellers. While, the incidence pattern in travellers returning from temperate countries closely mimicked the age pattern of indigenous Swedish cases, we noted that among travellers returning from tropical areas of Africa and Asia, the youngest children had twice as high risk as young adults, and more than four times the risk compared to older children [35]. This age pattern thus suggests other major routes of transmission than food or water, e.g. direct or indirect transmission from environmental sources. The flies would fit well in this concept.

#### 6. Dominance of solitary cases

If intake of chicken and undercooked meat (or cross-contamination from these food items) was a major route of transmission, clusters of cases within the same family should be common. Instead a striking feature of indigenous campylobacteriosis in Sweden is that the cases (except for in a few larger outbreaks) are solitary. A survey of notification data in one Swedish county over several years showed that it was exceptionally rare that cases shared the same address [37]. Information on the notification form indicating symptomatic cases around the notified patient was also very rare, even though this is specifically asked for. Solitary cases are instead more compatible with circumstance where an infected fly defecates on the plate of one family member, leaving the rest of the family unexposed.

### Testing the hypothesis

The fly hypothesis needs to be backed by further experimental and epidemiological studies. The best evidence would be if controlled intervention studies could show an effect on the incidence of *Campylobacter *infection by fly control, as has previously been done for shigellosis and diarrhoea [13-15]. Such studies could only be done in high incidence areas, and would require good laboratory support. In temperate regions such intervention studies would be less feasible. Instead, questions focusing on the exposure to flies, and possible nearby environmental *Campylobacter *sources, e.g. cattle farms, sewage treatment or fowls, should be included in forthcoming case-control studies on campylobacteriosis. This has been a neglected line of questioning so far. More data on the carriage of *Campylobacter *by flies in different settings where people could be exposed are also needed. An alternative, and more innovative, approach would be to combine information on the likely place/s of infection with data on environmental sources, in analytic studies using geographical information systems (GIS) tools.

## Conclusion

All the evidence in favour of the fly hypothesis is circumstantial and there may be alternative explanations to each of the findings supporting the hypothesis. However, in the absence of alternative explanations that could give better clues to the evasive epidemiology of *Campylobacter *infection, we believe it would be unwise to rule out flies as important mechanical vectors also of this disease.

## Summary

We put forward the hypothesis that flies play a more important role in the transmission of the bacteria, than has previously been recognized. Factors supporting this hypothesis are: 1) the low infective dose of *Campylobacter*; 2) the ability of flies to function as vectors; 3) a ubiquitous presence of the bacteria in the environment; 4) a seasonality of the disease with summer peaks in temperate regions and a more evenly distribution over the year in the tropics; 5) an age pattern for campylobacteriosis in western travellers to the tropics suggesting other routes of transmission than food or water; and finally 6) very few family clusters. The hypothesis should be further tested with experimental and epidemiological studies

## Competing interests

The author(s) declare that they have no competing interests.

## Authors' contributions

Bengt Normann raised the original idea and has studied the (lack of) family clustering. Yvonne Andersson contributed with in depth knowledge of campylobacteriosis. Karl Ekdahl did the literature search, looked into the seasonality and age distribution of the disease in domestic cases and returning travellers, and prepared the first draft of the manuscript. All authors have participated in revising the draft manuscript.

## Pre-publication history

The pre-publication history for this paper can be accessed here:


